# Lung Deposition of Inhaled Extrafine Beclomethasone Dipropionate/Formoterol Fumarate/Glycopyrronium Bromide in Healthy Volunteers and Asthma: The STORM Study

**DOI:** 10.1089/jamp.2021.0046

**Published:** 2022-08-04

**Authors:** Omar S. Usmani, Simonetta Baldi, Simon Warren, Ilaria Panni, Luca Girardello, François Rony, Glyn Taylor, Wilfried DeBacker, George Georges

**Affiliations:** ^1^ NHLI Imperial College London, London, United Kingdom.; ^2^ Chiesi Farmaceutici SpA, Parma, Italy.; ^3^ Cardiff Scintigraphics Ltd., Cardiff, United Kingdom.; ^4^ CROS NT Srl, Verona, Italy.; ^5^ Department of Respiratory Medicine, University of Antwerp, Antwerpen, Belgium.

**Keywords:** lung deposition, triple therapy, radiolabeling, peripheral airways, scintigraphy

## Abstract

**Background:**

An extrafine formulation triple therapy combination of beclomethasone dipropionate (BDP), formoterol fumarate (FF), and glycopyrronium bromide (GB) has been developed for the maintenance treatment of asthma and chronic obstructive pulmonary disease. This study used gamma scintigraphy to evaluate the intrapulmonary and extrapulmonary *in vivo* deposition of BDP/FF/GB, and the intrapulmonary regional distribution of the deposited formulation.

**Methods:**

This open-label uncontrolled nonrandomized single-dose study recruited 10 healthy volunteers and 9 patients with asthma. After a krypton-81m (^81m^Kr) ventilation scan was conducted, subjects inhaled study drug (four inhalations of BDP/FF/GB 100/6/12.5 μg radiolabeled using technetium-99 m [^99m^Tc]) through pressurized metered-dose inhaler, and a series of scintigraphic images were taken. The primary objective was to evaluate intrapulmonary drug deposition of BDP/FF/GB, determined as the percentage of nominal (i.e., metered) dose. Secondary endpoints included central/peripheral deposition ratio (C/P), and the standardized central/peripheral ratio (sC/P; ^99m^Tc aerosol C/P/^81m^Kr gas C/P).

**Results:**

All participants completed the study, with all scintigraphy procedures performed at one site. In patients with asthma, mean ± standard deviation intrapulmonary deposition was 25.50% ± 6.81%, not significantly different to that in healthy volunteers (22.74% ± 9.19%; *p* = 0.4715). Approximately half of the lung dose was deposited in the peripheral region of the lung (fraction deposited 0.52 ± 0.07 and 0.49 ± 0.06 in healthy volunteers and patients with asthma, respectively), resulting in C/P ratios of 0.94 ± 0.25 and 1.06 ± 0.25, respectively, with sC/P ratios of 1.80 ± 0.40 and 1.94 ± 0.38. Deposition patterns were similar in the two populations. BDP/FF/GB was well tolerated.

**Conclusions:**

This study confirmed that the extrafine particles delivered by BDP/FF/GB penetrate the peripheral areas of the lungs, with a similar proportion of particles deposited in the central and peripheral regions. Importantly, the deposition patterns were similar in healthy volunteers and patients with asthma, suggesting that disease characteristics are unlikely to impact drug deposition.

Clinical Trial Registration number: NCT03795350.

## Introduction

The pharmacological management of asthma centers on inhaled medication. The peripheral region of the lung contributes significantly to asthma's clinical impact, with inflammation or resistance in the peripheral lung correlating with symptoms, asthma control, health status, dyspnea, and exacerbations.^(1–6)^ Delivery and effective distribution of inhaled medication, not only to the central lung region but also to peripheral lung, is therefore important if treatment is to be optimized.^(7–9)^

*In vivo* gamma scintigraphy is conventionally used to assess lung deposition of an inhaled drug.^(10)^ The technique involves incorporation of a suitable radiolabel, for example, technetium-99 m (^99m^Tc), in such a way that the radiolabel acts as an accurate surrogate for the active pharmaceutical ingredients and does not alter the performance characteristics of the product. A previous gamma scintigraphy study evaluated pulmonary deposition of extrafine formulation inhaled corticosteroid (ICS)/long-acting β_2_-agonist (LABA) combination of beclomethasone dipropionate (BDP) plus formoterol fumarate (FF) delivered through pressurized metered dose inhaler (pMDI) in healthy volunteers and patients with asthma or chronic obstructive pulmonary disease (COPD).^(11)^ Extrafine particles (i.e., those with a mass median aerodynamic diameter <2 μm^(12)^) in aerosolized medicines are more able to consistently reach the peripheral lung region than nonextrafine particles,^(1,13,14)^ with resulting enhanced drug delivery to this area, and improved overall lung deposition.^(7)^ That previous study demonstrated good pulmonary deposition and homogeneous regional distribution of BDP and FF in the lungs, regardless of the disease state.

An extrafine triple therapy combination of BDP, FF, and the long-acting muscarinic antagonist (LAMA) glycopyrronium bromide (GB) has been developed for the maintenance treatment of asthma and COPD, delivered through pMDI. STORM (ScinTigraphic assessment Of tRiMbow lung deposition) was conducted to evaluate the intrapulmonary and extrapulmonary *in vivo* deposition of BDP/FF/GB pMDI using gamma scintigraphy, and the intrapulmonary regional distribution of the deposited formulation.

## Materials and Methods

### Trial design

This open-label uncontrolled nonrandomized single-dose study comprised a screening visit, 3–28 days before drug administration, and a treatment period during which subjects remained at the clinical site from the afternoon of Day –1 (the day before the study drug administration) until the morning of Day 2. At the screening visit, subject demographics, disease, medical, and smoking history, and spirometry results were recorded, and subjects were trained on correct use of the pMDI (using a placebo pMDI and an aerosol inhalation monitor). Subjects were also trained on correct pMDI use at the start of the treatment period, and again before administration of the study drug, and were instructed to use the same inspiratory flow for the corresponding steps during study drug inhalation.

During the treatment period, subjects fasted from 4 hours predose until 2 hours postdose, with fluid intake forbidden from 1 hour predose until 1 hour postdose, after which they were required to drink at least 240 mL of water every 2 hours for the following 6 hours. In addition, subjects were not permitted to lie down or sleep until 2 hours postdose, and were to avoid strenuous activities from 24 hours predose, and alcohol-containing food or beverages from 48 hours predose, both until the end of the treatment period.

At the start of the treatment period, a cobalt-57 (^57^Co) transmission scan was conducted to determine tissue attenuation correction factors [performed according to the methodology of Farr et al.^(15)^], with each subject undergoing a scan of the thorax (∼180 seconds) and of the head (∼60 seconds), using a flood field source. This was followed by a krypton-81m (^81m^Kr) ventilation scan to assess lung margins for the subsequent assessment of regional deposition, with subjects inhaling ^81m^Kr gas until a reading of 200,000 counts had been reached. Subjects then inhaled study drug, consisting of four inhalations of BDP/FF/GB 100/6/12.5 μg (total BDP/FF/GB dose 400/24/50 μg), through hydrofluoroalkane 134a pMDI, in a solution formulation. This was radiolabeled using ^99m^Tc-pertechnetate, not more than 2.5 MBq per actuation, prepared and validated as described in the Supplementary Data. Subjects inhaled study drug directly from the pMDI (with no spacer), while they were in an upright position, with breath held for 5–10 seconds before exhalation into a low resistance filter, with each subsequent inhalation to commence 30 seconds after the start of the previous inhalation. Subjects then washed their mouths out with ∼20 mL of water, with the washings collected, and then ate a small piece of bread, which was swallowed with ∼100 mL of water to clear radioactivity from the mouth and esophagus and concentrate extrapulmonary deposition within the stomach. The following scintigraphic images were then taken:

(1) Posterior and anterior images of lungs and of the stomach obtained simultaneously over 90 seconds, followed by
(2) lateral oropharynx region obtained over 60 seconds, followed by
(3) items external to the body of the subject: actuator, exhalation filter, mouthwash, and paper tissues.

Gamma camera imaging was performed using an Axis Dual Head gamma camera (Philips Medical Systems Limited, Cleveland, OH) with a field of view of 53 × 39 cm and low energy parallel hole collimators for ^99m^Tc and ^57^Co imaging, and medium energy parallel hole collimators for ^81m^Kr imaging. Images were processed using a commercially available nuclear medicine software package (Odyssey V9.4B; Philips Medical Systems Limited). Raw counts were corrected for radioactive decay, image duration, tissue attenuation and background radioactivity. The corrected recovered radioactive counts from each of the anatomical locations and from the mouthwash, exhalation filter, and the actuator were totaled to provide the metered dose.

Lung function was evaluated by spirometry, predose and up to 24 hours postdose, with blood sampled for the determination of plasma concentrations of BDP, beclomethasone 17-monopropionate (B17MP, an active metabolite of BDP), FF, and GB over a 24 hours period. Plasma samples were analyzed with validated liquid chromatography tandem mass spectrometry methods, with limits of quantification of 10, 20, 1, and 1 pg/mL for BDP, B17MP, FF, and GB, respectively. Adverse events were captured throughout the study.

The study was approved by the independent ethics committee at each institution, and by the Administration of Radioactive Substances Advisory Committee (ARSAC), and was performed in accordance with the principles of the Declaration of Helsinki and the International Conference on Harmonization notes for guidance on Good Clinical Practice (ICH/CPMP/135/95). The study was registered at ClinicalTrials.gov.

### Participants

Males or females with body mass index 18–32 kg/m^2^ were eligible. Healthy volunteers and patients with asthma were between the ages of 28–55 years, and were non- or ex-smokers (<5 pack-years, who stopped smoking >6 months before screening). The lung function of healthy volunteers was to be within normal limits (forced expiratory volume in 1 second [FEV_1_] ≥80% predicted normal, and ratio of FEV_1_ to forced vital capacity [FVC] >0.70); patients with asthma were diagnosed at least 12 months before entry (and before the age of 40 years), with prebronchodilator FEV_1_ 60%–80% predicted and reversibility of ≥12% and 200 mL within 30 minutes of inhalation of albuterol 400 μg.

Patients with asthma were excluded if they had received systemic corticosteroids within 4 weeks before screening, had nonpersistent asthma, or had a lower respiratory tract infection requiring antibiotics within 6 weeks before screening. Patients with COPD were also eligible, but due to difficulties in recruitment none entered before study recruitment was terminated due to the coronavirus disease 2019 (COVID-19) pandemic. All subjects provided written informed consent before any study-related procedure. Full inclusion and exclusion criteria are in the Supplementary Data.

The following medication was not to be used for the specified period before spirometry and gamma camera imaging: short-acting β_2_-agonists 6 hours; short-acting muscarinic antagonists 8 hours; LAMAs 48 hours; LABAs 24 hours (48 hours for ultra-LABAs such as indacaterol); ICSs 12 hours; leukotriene modifiers 72 hours; xanthine derivatives 72 hours.

### Outcomes

The primary objective was to evaluate the total intrapulmonary drug deposition of ^99m^Tc radiolabeled BDP/FF/GB, determined as the percentage of the nominal (i.e., metered, ex-valve) dose. The secondary objectives were to evaluate: central and peripheral lung drug distribution (as the ratio of central to peripheral deposition, the standardized central to peripheral ratio [sC/P, i.e., C/P ratio for ^99m^Tc aerosol/C/P ratio for ^81m^Kr gas], the fraction deposited in the central region and the fraction deposited in the peripheral region); extrathoracic drug deposition (percentage of nominal dose); amount of exhaled drug (percentage of nominal dose); amount of drug remaining in the actuator (percentage of nominal dose); BDP/B17MP, formoterol, and GB pharmacokinetics (area under the plasma concentration-time curve from 0 to the last quantifiable concentration [AUC_0–*t*_], to 30 minutes postdose [AUC_0–30 minutes_], and extrapolated to infinity [AUC_0–∞_], value of, and time to maximum and last observed plasma concentration [*C*_max_, *t*_max_, *C*_last_, and *t*_last_], and terminal plasma elimination half-life [*t*_½_]); efficacy, in terms of lung function assessments (FEV_1_, FVC, forced expiratory flow at 25%, 50%, and 75% [FEF_25_, FEF_50_ and FEF_75_]); and the correlation between lung deposition parameters and lung function parameters.

### Sample size and statistical methods

Since no data on lung deposition of BDP/FF/GB were available, and the primary objective was descriptive, no formal sample size calculation was performed. Ten subjects were to be enrolled in each group (healthy volunteers, asthma, and COPD), with the aim that at least eight per group completed all study procedures. This study size would be consistent with previous gamma scintigraphy lung deposition studies.^(11,16)^

The primary and secondary deposition variables were compared between groups using an analysis of variance (ANOVA) model including group as fixed effect. Other data are presented descriptively only. Adverse events were evaluated in the safety set, which was all subjects who received at least one dose of study drug. The primary and secondary deposition and lung function variables were analyzed in both the intention-to-treat (ITT) and the per-protocol (PP) populations; the ITT population was all subjects who received at least one dose of the study drug and with at least one available evaluation of efficacy after the baseline, whereas the PP population was all subjects from the ITT population without any major protocol deviations. The pharmacokinetic variables were analyzed in the pharmacokinetic population, which was all subjects from the safety population excluding subjects without any valid pharmacokinetic measurement or with major protocol deviations significantly affecting pharmacokinetics.

## Results

### Participants

The study ran from January 17, 2019 to April 3, 2020 (with recruitment terminated due to COVID-19) at two sites in the United Kingdom (all scintigraphy procedures were performed at one of these sites). A total of 10 healthy volunteers and 9 patients with asthma were recruited, all of whom completed the study. All were included in the ITT, safety, and pharmacokinetic populations; one of the patients with asthma was excluded from the PP population, as despite receiving asthma medication before the age of 40 years they were only formally diagnosed at the age of 42. Demographics were similar in the two populations (Table 1).

**Table 1.** Demographic Data

Parameter	Healthy volunteers (*N* = 10)	Patients with asthma (*N* = 9)
Age, years	39.1 ± 7.4 (28 to 49)	42.0 ± 6.2 (32 to 48)
Body mass index, kg/m^2^	25.2 ± 3.6 (21.4 to 31.0)	27.7 ± 2.7 (22.5 to 30.1)
Race		
Caucasian	10 (100)	8 (88.9)
Other	0 (0)	1 (11.1)
Gender		
Male	6 (60.0)	6 (66.7)
Female	4 (40.0)	3 (33.3)
FEV_1_^a^		
Absolute, L	3.84 ± 0.96 (2.72 to 5.46)	2.51 ± 0.45 (1.50 to 3.06)
% predicted	104.8 ± 9.4 (89 to 120)	70.9 ± 10.7 (54 to 88)
FEV_1_ reversibility, %^b^	—	17.4 ± 3.9 (13 to 24)
FVC, L^a^	4.93 ± 1.29 (3.33 to 7.64)	4.18 ± 1.06 (2.58 to 6.19)
FEV_1_/FVC ratio^a^	0.784 ± 0.086 (0.67 to 0.97)	0.616 ± 0.097 (0.40 to 0.74)
FEF_25_, L/sec^a^	7.27 ± 2.15 (4.53 to 10.45)	3.32 ± 0.91 (1.96 to 4.73)
FEF_50_, L/sec^a^	4.18 ± 1.39 (2.41 to 7.07)	1.80 ± 0.55 (0.92 to 2.38)
FEF_75_, L/sec^a^	1.56 ± 0.89 (0.66 to 3.77)	0.54 ± 0.17 (0.19 to 0.69)

Data are mean ± SD (range) or number (%).

^a^ Assessed at predose during the treatment period.

^b^ Assessed only in patients with asthma, with data either from patient records or assessed at screening.

FEF, forced expiratory flow; FEV_1_, forced expiratory volume in 1 second; FVC, forced vital capacity; SD, standard deviation.

### Outcomes

#### Drug deposition

**FIG. 1.**
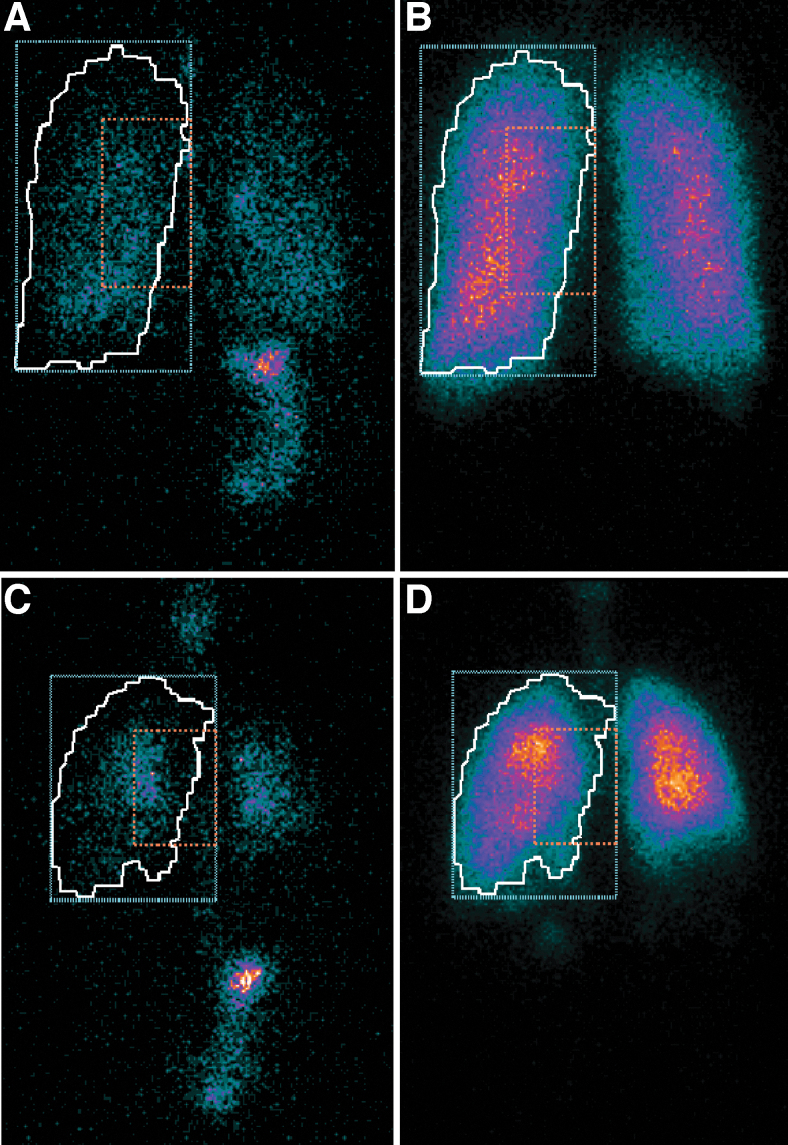
Example scans (reflected posterior views) from a healthy volunteer **(A** is lung deposition and **B** shows ventilation**)** and a patient with asthma **(**with **C** showing lung deposition and **D** showing ventilation**)**. The white line is the ventilated lung margin derived from the krypton-81m gas ventilation image (autocontour drawn by the nuclear medicine software that detects the margins of the lungs as those pixels with ∼10% of the maximum pixel count). The blue dotted line indicates the peripheral lung ROI, and the orange dotted line indicates the central lung ROI. The central and peripheral ROIs used here correspond to the inner and outer ROIs described in Newman et al.^(10)^ ROI, region of interest.

Example gamma scintigraphy scans of a healthy volunteer and a patient with asthma are shown in Figure 1.

Approximately 23% of the nominal dose of BDP/FF/GB was deposited in the lungs in healthy volunteers, compared with 26% in patients with asthma, with a very small proportion of the nominal dose exhaled (Table 2). On average half of the lung dose was deposited in the peripheral lung region (fraction deposited 0.52 and 0.49 in healthy volunteers and patients with asthma, respectively). Adjusting the mean aerosol C/P ratios (0.94 and 1.06, respectively) for the ^81m^Kr gas distribution to normalize for lung volume, the resultant mean sC/P values were 1.80 and 1.94. For all parameters, the adjusted mean differences (95% CI) between healthy volunteers and patients with asthma indicated similar deposition patterns in the two populations (Table 2). Results were consistent in the ITT and PP populations.

**Table 2.** Drug Deposition Parameters in the Two Populations, and Statistical Comparison Between Healthy Volunteers and Patients with Asthma (Intention-to-Treat Population)

Parameter	Healthy volunteers (*N* = 10)	Asthma (*N* = 9)	Adjusted mean difference (95% CI) [*p*]
Intrapulmonary drug deposition (% of nominal dose)	22.74 ± 9.19	25.50 ± 6.81	2.76 (−5.15, 10.67) [0.4715]
Aerosol (^99m^Tc) central to peripheral lung region ratio (C/P)	0.94 ± 0.25	1.06 ± 0.25	0.12 (−0.12, 0.36) [0.3024]
Ventilation gas (^81m^Kr) central to peripheral lung region ratio	0.52 ± 0.05	0.55 ± 0.10	0.04 (−0.04, 0.11) [0.3412]
Standardized central to peripheral ratio (sC/P)	1.80 ± 0.40	1.94 ± 0.38	0.13 (−0.24, 0.51) [0.4636]
Fraction of lung dose deposited in the central lung region	0.48 ± 0.07	0.51 ± 0.06	0.03 (−0.03, 0.09) [0.2929]
Fraction of lung dose deposited in the peripheral lung region	0.52 ± 0.07	0.49 ± 0.06	−0.03 (−0.09, 0.03) [0.2929]
Extrathoracic deposition (% of nominal dose)	61.60 ± 10.33	60.65 ± 7.16	−0.95 (−9.65, 7.76) [0.8210]
Amount of exhaled drug (% of nominal dose)	2.46 ± 1.27	2.03 ± 1.07	−0.42 (−1.57, 0.72) [0.4454]
Drug remaining in the actuator (% of nominal dose)	13.20 ± 2.14	11.81 ± 1.12	−1.39 (−3.07, 0.30) [0.1002]

Data for the individual populations are mean ± SD.

^99m^ Tc, technetium-99m; ^81m^Kr, krypton-81m.

### Lung function

As would be expected, the baseline (predose) lung function values were higher in healthy volunteers than patients with asthma (Table 1). In patients with asthma, there was an increase from baseline in mean FEV_1_ and FVC, with a peak at 30 minutes postdose (Supplementary Figs. S2 and S3); FEV_1_ remained above baseline for the full 24 hours assessment period. The initial FEV_1_ increase from baseline in healthy volunteers was lower, with no consistent effect on FVC. Similarly for the three FEF endpoints, BDP/FF/GB efficacy was generally greater in patients with asthma than healthy volunteers (Supplementary Figs. S4–S6). Results were consistent in the ITT and PP populations. In patients with asthma, there were no significant correlations between drug deposition and any of the baseline lung function parameters.

### Pharmacokinetics

Although the pharmacokinetic parameters were not formally statistically compared between the two populations, there did not appear to be any obvious differences in BDP, B17MP, or FF values between patients with asthma and healthy volunteers (Supplementary Table S1). For GB, there appeared to be a trend toward slightly higher systemic exposure (*C*_max_, *C*_last_, AUC_0-30 minutes_, and AUC_0-*t*_) in patients with asthma compared with healthy volunteers.

### Safety

No adverse events were reported in the healthy volunteers. Two patients with asthma reported adverse events (medical device site bruise and headache), both mild in severity, nonserious, and not related to study drug. There were no deaths in the study, and no adverse events led to withdrawal.

## Discussion

This is the first study to evaluate lung deposition of the inhaled triple combination of BDP/FF/GB through pMDI by means of gamma scintigraphy. We included subjects with and without airflow obstruction: In both healthy volunteers and patients with airflow obstruction (i.e., asthma), ∼25% of the nominal (metered) dose of extrafine BDP/FF/GB was deposited in the lungs, with a similar proportion of the lung dose deposited in the peripheral and central regions. Importantly, the presence of asthma did not impact any of the deposition parameters, with no significant differences between the two populations.

Standardizing the C/P ratio (by dividing the C/P ratio by the results of the ^81m^Kr ventilation scan) corrects for lung volume and so facilitates comparisons with other studies using similar scintigraphic methods and regions of interest.^(17)^ The results of this study are consistent with those of a previous gamma scintigraphy study that evaluated lung deposition of the inhaled ICS/LABA combination of extrafine BDP/FF in eight healthy volunteers and eight patients with asthma, and that defined the central and peripheral regions of interest in the same way as this study.^(11)^ In that study, the sC/P ratios were 1.42 and 1.96 in healthy volunteers and patients with asthma, respectively, with drug distribution throughout the lung in both populations. In the current study, the sC/P ratios were 1.80 and 1.94, respectively, with drug again distributed throughout the lung. As with the BDP/FF study, the regional lung distribution parameters, that is, the fraction of aerosol deposited in the central and peripheral lung regions, C/P, and sC/P ratios, suggest that the extrafine particles generated from this BDP/FF/GB formulation penetrate the lung and deposit predominantly by sedimentation throughout the large and small airways.^(18)^

Difficulties in recruitment (in particular due to the COVID-19 pandemic) meant that no patients with COPD had entered the study. However, the previous BDP/FF study was able to recruit the planned eight patients with COPD; the deposition patterns were similar in these patients to those with asthma or the healthy volunteers.^(11)^ Given the similarity of the results in the two studies for the healthy volunteer and asthma populations, BDP/FF/GB would also be expected to distribute throughout the central and peripheral lung regions of patients with COPD.

Although planar gamma scintigraphy is the most common (and recognized) method to investigate lung deposition of inhaled medication, a disadvantage is that it is a two-dimensional method. As a consequence, if small and large airways overlap on the image, planar scintigraphy cannot distinguish between them, such that molecules may be considered to deposit centrally when they have actually deposited in the peripheral region and *vice versa*. A more recent development is functional residual imaging (FRI), which combines aerosol delivery performance profiles, patients' high-resolution computed tomography lung scans, and patient-derived inhalation profiles to simulate three-dimensional aerosol lung deposition. Two previous studies have used FRI to model the three-dimensional deposition of BDP/FF/GB, one in comparison with nonextrafine formulation fluticasone furoate/vilanterol/umeclidinium,^(19)^ and the other in comparison with extrafine BDP/FF,^(20)^ both of which demonstrated high peripheral deposition of BDP/FF/GB.

The lung function data confirm the bronchodilator efficacy of BDP/FF/GB in patients with asthma. Even though patients inhaled a single dose, and BDP/FF/GB is administered twice daily, FEV_1_ remained above baseline for the full 24-hour assessment period. Importantly, however, baseline lung function in these patients did not correlate with any of the drug deposition parameters, further supporting the lack of impact of disease characteristics on deposition. Furthermore, systemic exposure of B17MP, BDP, and FF was similar in the two populations. There was a trend toward higher systemic exposure of GB in patients with asthma compared with healthy volunteers. This could potentially be due to the changes in the airway epithelium frequently observed in patients with asthma,^(21–23)^ thereby increasing tissue permeability. Importantly, however, only two patients with asthma (and no healthy volunteers) experienced adverse events, both mild in severity, and not related to study drug.

## Conclusions

The study confirmed that the extrafine particles delivered by BDP/FF/GB were able to penetrate the peripheral region of the lung, with a similar proportion of particles deposited in the central and peripheral regions. This is a desirable feature for an inhaled triple therapy that targets receptors that are dispersed throughout the airways.^(24)^ Importantly, the deposition patterns were similar in the healthy volunteers and patients with asthma, suggesting that disease characteristics are unlikely to impact drug deposition.

## Data Availability Statement

Chiesi commits to sharing with qualified scientific and medical researchers, conducting legitimate research, the anonymized patient-level and study-level data, the clinical protocol and the full clinical study report of Chiesi Farmaceutici SpA-sponsored interventional clinical trials in patients for medicines and indications approved by the European Medicines Agency and/or the U.S. Food and Drug Administration after January 1, 2015, following the approval of any received research proposal and the signature of a Data Sharing Agreement. Chiesi provides access to clinical trial information consistently with the principle of safeguarding commercially confidential information and patient privacy. Other information on Chiesi's data sharing commitment, access, and research request's approval process are available in the Clinical Trial Transparency section of http://www.chiesi.com/en/research-and-development/.

## Supplementary Material

Supplemental data

## Notes

Reviewed by:

Steven Newman

Federico Lavorini
