# Supplementary material for: Lung Deposition of Inhaled Extrafine Beclomethasone Dipropionate/Formoterol Fumarate/Glycopyrronium Bromide in Healthy Volunteers and Asthma: The STORM Study
Source: J Aerosol Med Pulm Drug Deliv. 2022 Aug 4;35(4):179–85. doi: 10.1089/jamp.2021.0046 (PMC9416540; doi:10.1089/jamp.2021.0046)
Supplement: Supplemental data [file Supp_Data.pdf]

Lung deposition of inhaled extrafine beclomethasone dipropionate/formoterol fumarate/glycopyrronium bromide in healthy volunteers and asthma: The STORM study

Omar S Usmani, Simonetta Baldi, Simon Warren, Ilaria Panni, Luca Girardello, François Rony, Glyn Taylor, Wilfried DeBacker, George Georges

Supplement

Methods

Inclusion criteria

	Healthy	Asthma	COPD
1. Subject's written informed consent obtained prior to any study-related procedure.	✓	✓	✓
2. Male and female subjects aged 28–55 years inclusive.	✓	✓	
3. Male and female patients aged 40–80 years inclusive.			✓
4. Ability to understand the study procedures, the risks involved and ability to demonstrate correct use of the inhaler using the AIM™ (Aerosol Inhalation Monitor) Vitalograph®.	✓	✓	✓
5. Body mass index (BMI) between 18 and 32 kg/m^2 extremes inclusive.	✓	✓	✓
6. Non- or ex-smokers who smoked <5 pack years (pack-years = the number of cigarette packs per day times the number of years) and stopped smoking >6 months prior to screening.	✓	✓	
7. A smoking history of at least 10 pack-years.			✓
8. Current or ex-smokers were eligible. Previous smokers are defined as those who have stopped smoking for at least 6 months prior to screening visit, with smoking cessation at least 3 months prior to the screening visit.			✓
9. Good physical status, determined on the basis of the medical history and a general clinical examination, at screening.	✓	✓	✓
10. Vital signs within normal limits.	✓	✓	✓
11. 12-lead digitized electrocardiogram (12-lead ECG) considered as normal at screening and at Day –1 (40≤ heart rate ≤100 bpm, 120 msec ≤ PR ≤ 200 msec, QRS ≤120 msec, QTcF ≤450 msec for males and QTcF ≤470 msec for females); (Note: In case of abnormal ECG at screening, the test could be repeated once before Day 1).	✓		
12. 12-lead ECG considered as normal at screening and Day –1 (40≤ heart rate ≤100bpm, 120 msec ≤ PR ≤220 msec, QRS ≤120 msec, QTcF ≤450 msec for males and QTcF ≤470 msec for females); (Note: In case of abnormal ECG, the test could be repeated once before Day 1).		✓	

	Healthy	Asthma	COPD
13. 12-lead ECG considered as normal at screening and Day –1 (40 ≤ heart rate ≤110bpm, 120 msec ≤ PR ≤220 msec, QRS ≤120 msec, QTcF ≤450 msec for males and QTcF ≤470 msec for females); (Note: In case of abnormal ECG at screening, the test could be repeated once before Day 1).			✓
14. Lung function measurements within normal limits at screening: FEV_1 ≥80% of predicted normal and FEV_1/FVC >0.70; (Note: in case of abnormal lung function measurement results, the test could be performed again once before Day 1).	✓		
15. Diagnosis of asthma: Established diagnosis of permanent asthma for at least 12 months according to Global Initiative for Asthma guidelines. Diagnosis should have been done before the patient's age of 40 year and appropriately documented at screening visit, based on medical documentation and medical history.		✓	
16. Patients with a pre-bronchodilator 60% ≤ FEV_1 <80% of predicted normal after appropriate wash-out from bronchodilators. (Note: in case of abnormal lung function measurement results, the test could be performed again once before Day 1).		✓	
17. Patients with a documented reversibility defined as an increase ≥12% and 200 mL over baseline within 30 min after inhalation of 400 µg albuterol pMDI. The documented test had to be performed no more than 12 months before screening or at screening. (Note: in the latter case, if the reversibility threshold is not met at screening, the test could be repeated once before Day 1).		✓	
18. Established diagnosis of COPD (according to Global Initiative for Chronic Obstructive Pulmonary Disease guidelines) at least 12 months before the screening visit.			✓
19. A post-bronchodilator FEV_1 ≤50% of predicted normal and a post-bronchodilator FEV_1/FVC <0.7 10–15 minutes after 4 puffs (4x100 µg) of albuterol pMDI. (Note: if the criterion is not met at screening, the test can be repeated before Day1).			✓

	Healthy	Asthma	COPD
20. Males fulfilling one of the following criteria:<br>a. Males with non-pregnant women of childbearing potential (WOCBP) partners: they and/or their partner of childbearing potential must have used a highly effective birth control method in addition to the male condom from the signature of the informed consent and until 90 days after the follow-up visit. Subjects must not have donated sperm during the study and for 90 days after the follow-up visit or<br>b. Males with pregnant WOCBP partner: they must have used male contraception (condom) from the signature of the informed consent and until 90 days after the follow-up visit. Subjects must not have donated sperm during the study and for 90 days after the follow-up visit or<br>c. Non-fertile male subjects (contraception was not required in this case) or<br>d. Males with partner not of childbearing potential (contraception was not required in this case).	✓	✓	✓
21. WOCBP fulfilling one of the following criteria:<br>a. WOCBP with fertile male partners: they and/or their partner must have used a highly effective birth control method with low user dependency from the signature of the informed consent and until 30 days after the follow-up visit or<br>b. WOCBP with non-fertile male partners (contraception was not required in this case).	✓	✓	✓
22. Females of non-childbearing potential defined as physiologically incapable of becoming pregnant (i.e. post-menopausal or permanently sterile). Tubal ligation or partial surgical interventions were not acceptable.	✓	✓	✓

Exclusion criteria

	Healthy	Asthma	COPD
1. Pregnant or lactating women.	✓	✓	✓
2. Participation to another clinical trial where investigational drug was received and last investigations were performed less than 90 days prior to screening.	✓	✓	✓

	Healthy	Asthma	COPD
3. Clinically relevant and uncontrolled respiratory, cardiac, hepatic, gastrointestinal, renal, endocrine, metabolic, neurologic, or psychiatric disorders that may interfere with successful completion of this protocol.	✓	✓	✓
4. Clinically relevant abnormal laboratory values at screening suggesting an unknown disease and requiring further clinical investigation or which may impact the safety of the subject or the evaluation of the result of the study according to the Investigator's judgment. (Note: in case of abnormal laboratory values, that could indicate a temporary condition, the test could have been performed again once, with the results available prior to Day 1).	✓	✓	✓
5. Subjects with medical diagnosis of narrow-angle glaucoma, prostatic hypertrophy or bladder neck obstruction that in the opinion of the investigator would prevent use of anticholinergic.	✓	✓	✓
6. Subjects with history of breathing problems (i.e. history of asthma including childhood asthma).	✓		
7. Positive HIV1 or HIV2 serology at screening.	✓	✓	✓
8. Blood donation or blood loss (equal or more than 450 mL) less than 2 months prior to screening or prior to Day 1.	✓	✓	✓
9. Documented history of alcohol abuse within 12 months prior to screening or a positive alcohol breath test at screening or prior to Day 1.	✓	✓	✓
10. Documented history of drug abuse within 12 months prior to screening or a positive urine drug screen evaluated at screening or prior to Day 1 unless it could explained by the subject medication. (Note: in case of abnormal laboratory values, that could indicate a temporary condition, the test could have been performed again once, with the results available prior to Day 1. Urine drug test could be repeated once within 4 hours or in a separate visit to avoid false positive).	✓	✓	✓
11. Positive results from the hepatitis serology indicating acute or chronic Hepatitis B or Hepatitis C at screening (i.e. positive HB surface antigen (HBsAg), HB core antibody (IgM anti-HBc), HC antibody).	✓	✓	✓

	Healthy	Asthma	COPD
12. Subjects with cardiovascular conditions such as, but not limited to unstable ischemic heart disease, NYHA Class III/IV left ventricular failure, acute ischemic heart disease in the year prior to study screening, which may impact the safety of the subject or the evaluation of the result of the study according to the Investigator's judgment.	✓	✓	✓
13. Positive urine test for cotinine at screening or prior Day 1 (Note: in case of abnormal values, the test could be performed again once, with the results available prior to Day 1).	✓	✓	
14. Unsuitable veins for repeated venipuncture/cannulation.	✓	✓	✓

Radiolabeling of study drug

Methods

^{99m}Tc pertechnetate eluent from a commercial generator was processed to reduce the sodium chloride (NaCl) content, and was reconstituted in a small volume of absolute ethanol (PhEur). The ^{99m}Tc was introduced into the pMDI formulation using a ‘cold’ transfer method, which was performed at controlled humidity (<20% relative humidity) in a glove box. The ^{99m}Tc, in a small volume of ethanol, was dispensed into a new, clean, pre-cooled, receiving canister. A second canister containing the test formulation was cooled by placing in a mixture of dry ice and ethanol, when sufficiently cold the metering valve was removed and the contents were rapidly transferred to the canister containing the radiolabel and a new valve was immediately crimped into place. Following crimping of the new valve the canister was mechanically shaken for 10 min. Cold transfer efficiency was determined gravimetrically and in all cases exceeded 85%. The introduction of the radiolabel in this way was considered appropriate, as ethanol is an excipient in the test product which is formulated as a solution of the active ingredients in propellant hydrofluoroalkane 134a.

Initial experiments were conducted in the absence of ^{99m}Tc, i.e., ‘cold radiolabeling’, in order to confirm that the transfer process did not affect the performance of the product. Moisture levels in the ‘cold labelled’ canisters were measured using a Karl Fischer titrator. The cold labelled formulations were characterized in terms of aerodynamic particle size distribution (using a Next Generation Impactor) and delivered dose (using a Dosage Unit Sampling Apparatus) and compared to control canisters from the same batch. The acceptance criteria were that the emitted (metered) dose, fine particle fraction (FPF), and the fine particle mass (FPM) of the active pharmaceutical ingredients (APIs) from control and ‘cold labelled’ canisters should not differ by more than 15%.

Upon successful completion of cold labelling experiments, the same technique was used to introduce ^{99m}Tc into the formulations. For these assessments an additional acceptance criterion was introduced, such that the FPF of ^{99m}Tc should not differ by more than 15% from

the FPF of the APIs from radiolabeled products. The emitted dose of ^{99m}Tc at the reference time was targeted to 5–10 MBq, i.e. not more than 2.5 MBq / actuation. Initial experiments were conducted using low levels of ^{99m}Tc before repeating at levels to be used in clinical assessments. Finally, three validation batches were produced using the approved Master Batch Records before the clinical phase commenced.

During validation, the stability of the radiolabeled products was assessed over a time period relevant to the clinical procedures, with delivered dose and aerosol performance determined as soon as possible after radiolabeling and again at 4 h post radiolabeling, i.e., the anticipated clinical dosing period.

Due to the short physical half-life of ^{99m}Tc (approximately 6 h) radiolabeled product was prepared on the morning of each dosing day. In all, seven radiolabeled batches were prepared for clinical dosing. Quality control measures were performed on each dosing day to characterize the emitted dose and FPF for ^{99m}Tc prior to product release and dosing.

Validation

Cold transfer did not affect the delivered dose or aerosol characteristics of the formulation, and moisture levels in cold transfer and radiolabeled canisters was acceptable. In addition, all development batches and validation batches complied with acceptance criteria and all seven clinical batches complied with acceptance criteria. Example drug and radiolabel distribution for radiolabeled batches and control is shown in Supplementary Figure 1. There were small differences in the recovery of radiolabel and drugs from the Induction Port (IP), and the filter. In the case of the IP the mean difference between ^{99m}Tc and the recovered drugs was approximately 10%. For the filter recovery the mean difference between ^{99m}Tc and the recovered drugs was approximately 4%. Considering ^{99m}Tc was used as a surrogate for the three active pharmaceutical ingredients, the minimum difference in recovery from the IP was approximately 8% (^{99m}Tc versus BDP) and in the filter was approximately 3% (^{99m}Tc versus BDP). These differences are unlikely to have influenced the overall results.

Supplementary Figure 1. Summary of aerodynamic particle size distribution of radiolabeled active pharmaceutical ingredients and technetium-99m (n=3 radiolabeled and 3 control canisters) determined using Next Generation Impactor.

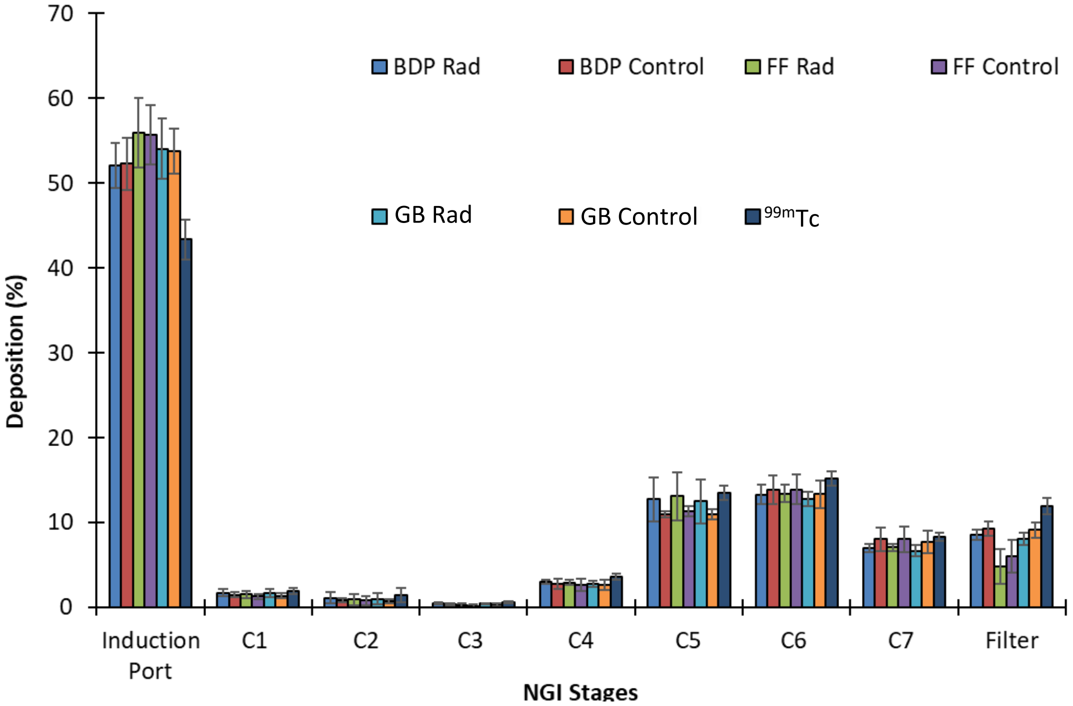

Data are mean and standard deviation. BDP, beclomethasone dipropionate; FF, formoterol fumarate; GB, glycopyrronium bromide; ^{99m}Tc, technetium-99m.

Results

Supplementary Figure 2. Mean FEV_1 change from baseline (ITT population).

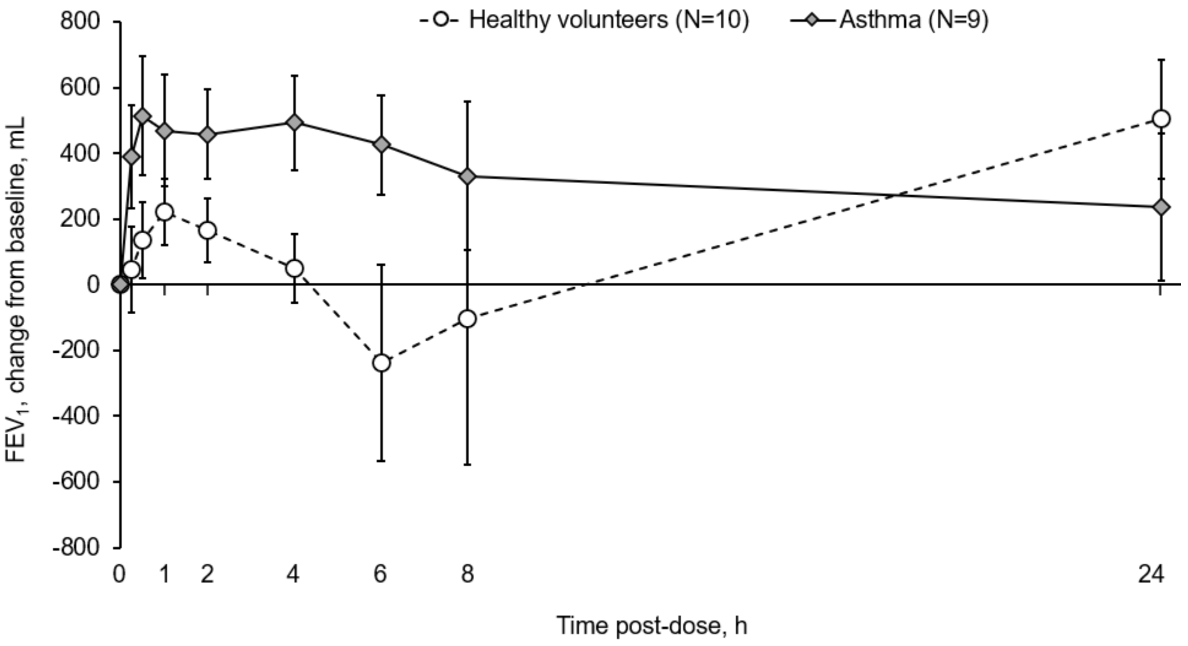

Data are mean and 95% confidence interval. FEV_1, forced expiratory volume in 1 sec; ITT, intention-to-treat.

Supplementary Figure 3. Mean FVC change from baseline (ITT population).

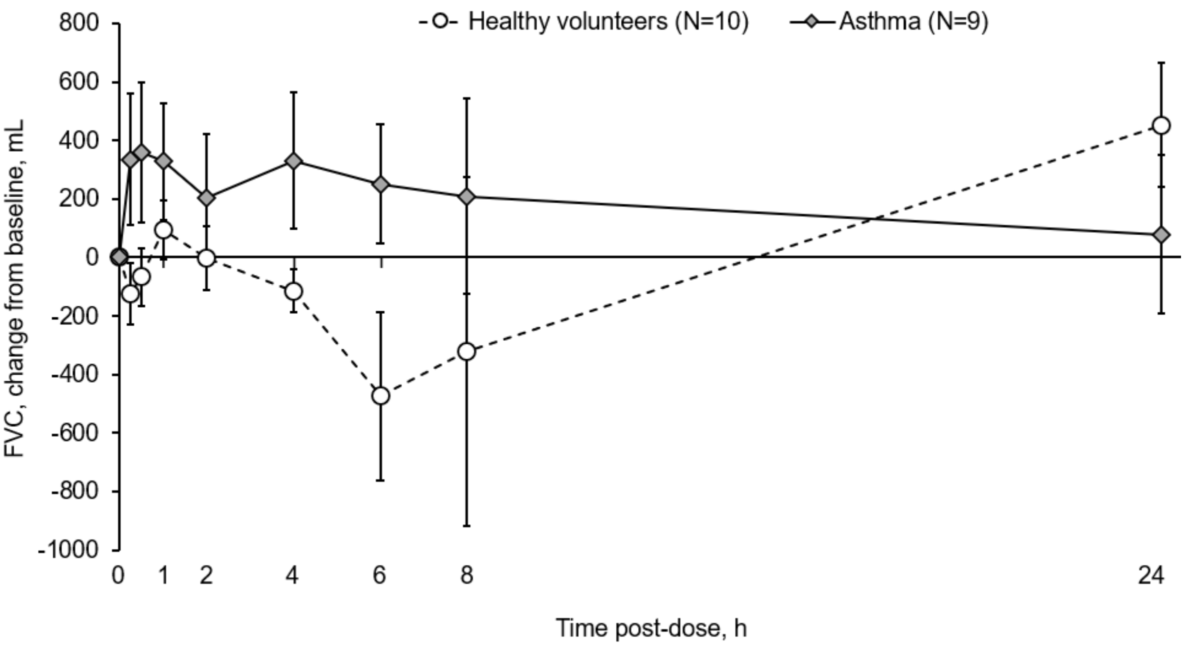

Data are mean and 95% confidence interval. FVC, forced vital capacity; ITT, intention-to-treat.

Supplementary Figure 4. Mean FEF_{25} change from baseline (ITT population).

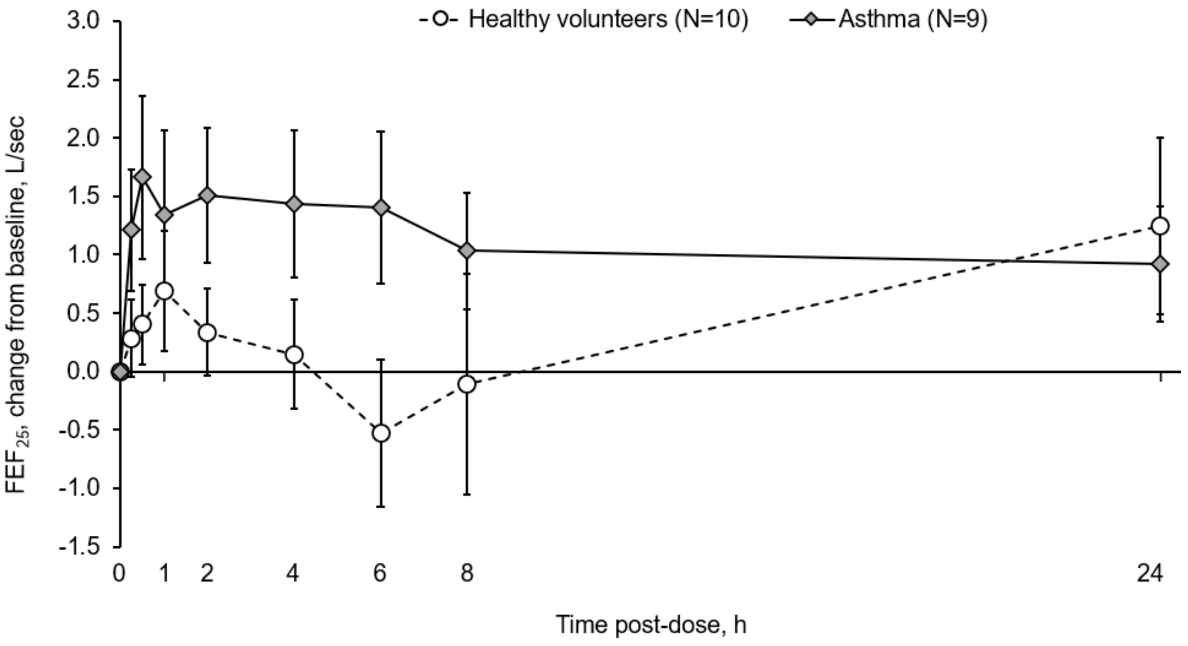

Data are mean and 95% confidence interval. FEF, forced expiratory flow; ITT, intention-to-treat.

Supplementary Figure 5. Mean FEF_{50} change from baseline (ITT population).

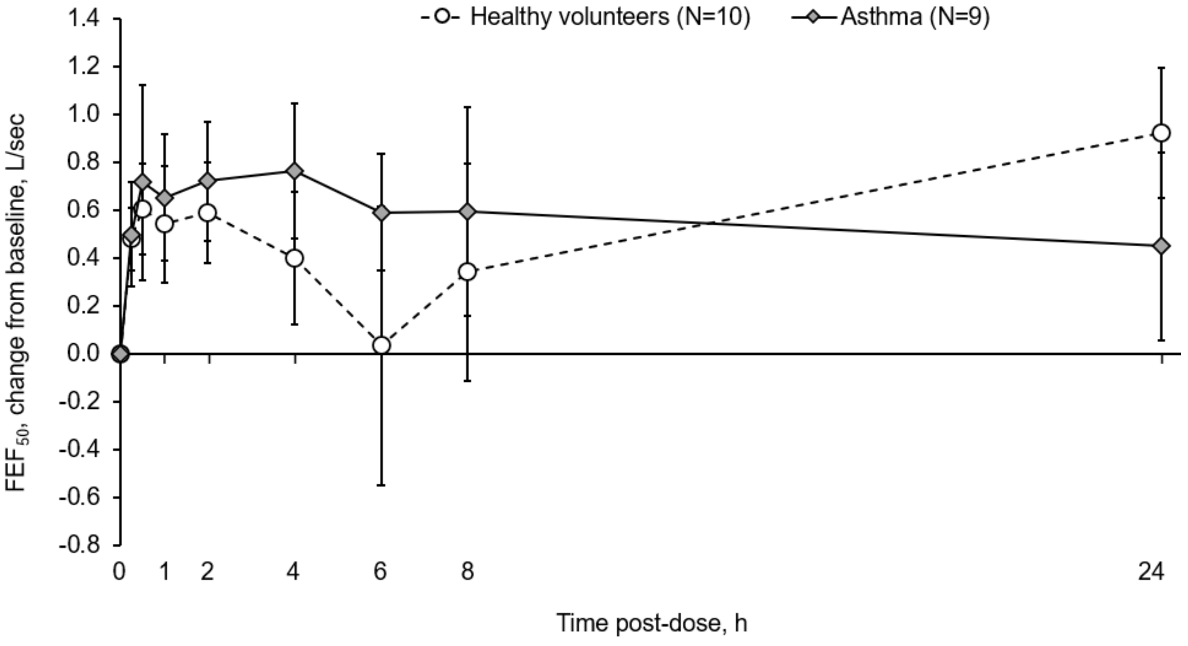

Data are mean and 95% confidence interval. FEF, forced expiratory flow; ITT, intention-to-treat.

Supplementary Figure 6. Mean FEF_{75} change from baseline (ITT population).

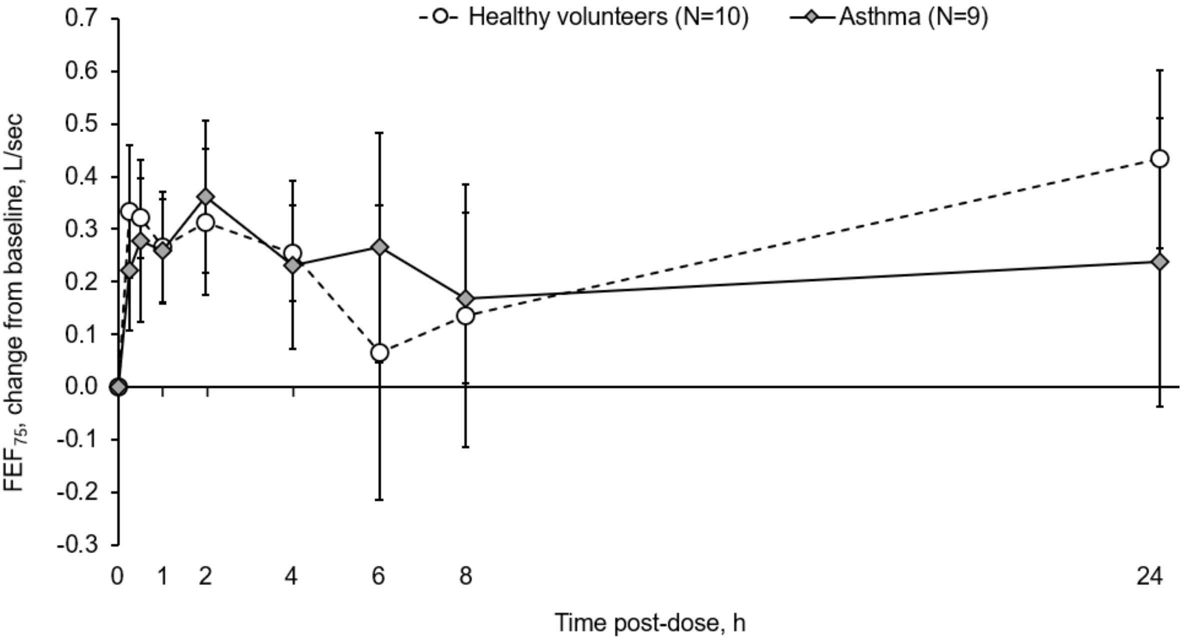

Data are mean and 95% confidence interval. FEF, forced expiratory flow; ITT, intention-to-treat.

Supplementary Table 1. Pharmacokinetic parameters following inhalation of a single dose of ^{99m}Tc radiolabeled BDP/FF/GB (pharmacokinetic population).

	C_{max} (pg/mL)	t_{max} (h)	C_{last} (pg/mL)	t_{last} (h)	$AUC_{0-30min}$ (h*pg/mL)	AUC_{0-t} (h*pg/mL)	$AUC_{0-\infty}$ (h*pg/mL)	$t_{½}$ (h)
BDP								
Healthy volunteers (N=10)	422± 188[a]	0.17[a] (0.17, 0.17)	19.2± 8.42	1.0 (0.5, 1.0)	N/A	101± 47.6[a]	N/A	N/A
Asthma (N=9)	484± 195	0.17 (0.17, 0.17)	21.6± 10.7	1.0 (0.5, 2)	N/A	118± 56.0	N/A	N/A
B17MP								
Healthy volunteers (N=10)	700± 238[a]	0.5[a] (0.17, 1.0)	53.0± 20.3	12 (12, 12)	246± 98.3[a]	2747± 940[a]	3041± 1051[a]	3.80 (2.77, 5.44)
Asthma (N=9)	852± 327	0.5 (0.25, 1.0)	59.6± 16.2	12 (12, 12)	287± 115	3026± 537	3372± 611	3.64 (2.92, 8.31)
Glycopyrronium bromide								
Healthy volunteers (N=10)	21.4± 11.5[a]	0.5[a] (0.17, 0.5)	2.59± 1.00	24 (24, 24)	7.28± 3.70[a]	90.9± 29.9[a]	N/R	N/R
Asthma (N=9)	36.1± 15.3	0.5 (0.17, 0.5)	2.63± 0.805	24 (24, 24)	12.6± 4.97	133± 47.6	N/R	20.2[b] (14.6, 51.4)
Formoterol fumarate								
Healthy volunteers (N=10)	29.9± 8.99[a]	0.17[a] (0.17, 0.17)	1.45± 0.462	24 (8, 24)	9.66± 2.32[a]	90.2± 25.2[c]	112± 24.4[d]	8.30[a] (5.00, 12.0)
Asthma (N=9)	32.4± 13.0	0.17 (0.17, 0.25)	1.59± 0.455	24 (12, 24)	11.4± 4.33	109± 32.3	129± 24.1	8.98 (3.64, 13.6)

Data are mean±SD, except for except for t_{max}, t_{last}, and $t_{½}$ which are median (minimum, maximum). [a]N=9, [b]N=6, [c]N=8, [d]N=7.

C_{max}, maximum plasma concentration; t_{max}, time to maximum plasma concentration; C_{last}, last observed plasma concentration; t_{last}, time to last observed plasma concentration; AUC, area under the plasma concentration-time curve from 0 to 30 min post-dose ($AUC_{0-30min}$), to the last quantifiable concentration (AUC_{0-t}), and extrapolated to infinity [$AUC_{0-\infty}$]; $t_{½}$, terminal plasma elimination half life; N/A, not assessed; N/R, not reported (all patients had $AUC_{0-\infty}$ >20% and/or adjusted R squared <0.85).
